# Visual Search in 3D: Effects of Monoscopic and Stereoscopic Cues to Depth on the Validity of Feature Integration Theory and Perceptual Load Theory

**DOI:** 10.3389/fpsyg.2021.596511

**Published:** 2021-03-17

**Authors:** Ciara M. Greene, John Broughan, Anthony Hanlon, Seán Keane, Sophia Hanrahan, Stephen Kerr, Brendan Rooney

**Affiliations:** School of Psychology, University College Dublin, Dublin, Ireland

**Keywords:** visual search, 3D, stereoscopic depth, monoscopic depth, feature integration, distractor congruency, perceptual load theory

## Abstract

Previous research has successfully used feature integration theory to operationalise the predictions of Perceptual Load Theory, while simultaneously testing the predictions of both models. Building on this work, we test the extent to which these models hold up in a 3D world. In two experiments, participants responded to a target stimulus within an array of shapes whose apparent depth was manipulated using a combination of monoscopic and stereoscopic cues. The search task was designed to test the predictions of (a) feature integration theory, as the target was identified by a single feature or a conjunction of features and embedded in search arrays of varying size, and (b) perceptual load theory, as the task included congruent and incongruent distractors presented alongside search tasks imposing high or low perceptual load. Findings from both experiments upheld the predictions of feature integration theory, regardless of 2D/3D condition. Longer search times in conditions with a combination of monoscopic and stereoscopic depth cues suggests that binding features into three-dimensional objects requires greater attentional effort. This additional effort should have implications for perceptual load theory, yet our findings did not uphold its predictions; the effect of incongruent distractors did not differ between conjunction search trials (conceptualised as high perceptual load) and feature search trials (low perceptual load). Individual differences in susceptibility to the effects of perceptual load were evident and likely explain the absence of load effects. Overall, our findings suggest that feature integration theory may be useful for predicting attentional performance in a 3D world.

## Introduction

In order to be useful, cognitive theories must be simplified models of a complex world, from which we can derive specific predictions. In order to be meaningful, these models must also be scalable and applicable to real world situations. Models of visual search are principally based around perception of two-dimensional (2D) stimuli such as shapes on a computer screen, but are used to make inferences about our interaction with the three-dimensional (3D) world around us. When attempting to test the ecological validity of such models, researchers typically add complexity in a controlled manner, one variable at a time. In this paper we attempt to scale two models of visual search – Treisman’s feature integration theory and Lavie’s perceptual load theory– by incorporating monoscopic and stereoscopic cues to depth into an existing paradigm in which both models can be tested simultaneously. Both feature integration theory and perceptual load theory are simplified models of the type described above, from which useful predictions about the world have been generated. By the simple addition of a third dimension we literally add depth to the models, and test their validity in a three-dimensional world.

### Feature Integration Theory

Feature integration theory (Treisman and Gelade, 1980; Treisman, 1988) is a model of visual attention in which perception occurs at the level of features rather than objects; once perceived, attentional resources are required to bind features into coherent objects. While feature-level perception can and does occur in parallel, the integration of features into objects occurs serially. The classic finding in feature integration theory is therefore that reaction times for identifying objects on the basis of a single feature (e.g., colour) are relatively unaffected by the number of distractor items in the search array. In contrast, when items must be identified on the basis of a conjunction of features (e.g., colour and shape) each item in the array must be processed in turn and reaction time to identify the target will therefore increase linearly with the number of items in the array (Treisman and Gelade, 1980). Feature integration theory has been supported by a plethora of research (see Quinlan, 2003; Humphreys, 2016 for reviews), and while it has been partially subsumed into more comprehensive theories (e.g., Duncan and Humphreys, 1989; Desimone and Duncan, 1995), it has undeniably made a significant contribution to our understanding of visual attention. While early work using this model was largely restricted to analysis of basic features such as colour and shape, subsequent work has included dynamic features such as direction of motion (Dick et al., 1987; Thornton and Zdravković, 2020) and axis of rotation (Schill et al., 2020). Recently, feature integration theory has been particularly useful in the development of a concrete operationalisation of perceptual load.

### Perceptual Load Theory

The premise of perceptual load theory (Lavie, 1995; Lavie et al., 2004) is that perceptual capacity is limited, and that perceptual processing continues automatically until that capacity is filled. If a task or scene imposes high perceptual load then, according to the model, all available capacity will be used up in processing the central stimuli and no resources will be available to process additional distractor stimuli. In contrast, if the task imposes a low level of perceptual load, leftover attentional resources will “spill over” and process peripheral or distractor stimuli. Thus, perceptual load theory predicts that distractor perception will be reduced or eliminated under high perceptual load. This is typically assessed using flanker tasks in which a peripheral distractor stimulus requires a response that is either congruent or incongruent with the response to the target stimulus (Lavie, 2005). For example, a typical paradigm requires participants to search an array of letters and report the presence of either an X or an N. The search array is flanked by a distractor stimulus that may be response-congruent (e.g., an X when the target is an X) or response-incongruent (an X when the target is an N). Distractor processing is indicated by longer reaction times on incongruent trials because response competition between the target and distractor must be resolved before a response can be made. While a substantial congruency effect is expected under low perceptual load, this is predicted to be reduced or eliminated under high load. In this standard paradigm, perceptual load is manipulated by varying the features of the non-target items in the search array; in the low perceptual load condition, the target is presented in an array of O’s while in the high load condition the non-target items are angular letters such as M, H and Z.

Although perceptual load theory has been influential over the past twenty years, the concept of perceptual load has been criticised for its vagueness and lack of a clear operational definition (Murphy et al., 2016). Murphy and Greene (2017b) recently described a series of experiments demonstrating that the classic comparison in feature integration theory – between feature and conjunction searches – could act as an operationalisation of perceptual load theory. The five experiments reported in that paper tested the predictions of both feature integration theory and perceptual load theory within a single paradigm, and found that both were upheld. Following Lavie (1995), Murphy and Greene noted that feature and conjunction searches could be conceived of as low and high perceptual load tasks, respectively. In the most basic form of their paradigm, participants search a visual array for a specific target (a red triangle) and report its orientation while ignoring a distractor triangle whose orientation may be congruent (i.e., both pointing up) or incongruent with the target (one pointing up, one pointing down). The target is contained in a search array of between 2 and 10 items from which the target can be discriminated on the basis of one feature (e.g., a red triangle in a field of yellow triangles) or on the basis of a conjunction of features (e.g., a red triangle in a field of yellow triangles and red or yellow squares). They observed that response times in this paradigm followed the typical pattern expected of feature vs. conjunction searches, with fast responses to feature searches irrespective of the number of distractors, and slower responses to conjunction searches that required a serial inspection of each item in the array. The reaction time data also fit the predictions of perceptual load theory, with reduced distractor congruency effects under high load compared with low load. Subsequent experiments found similar results with this paradigm under a range of different conditions in which the stimulus presentation time, distractor location and stimulus complexity were varied, leading incrementally toward a more ecologically valid design. It has been suggested that the reduction in distractor interference under high load conditions may sometimes be caused by the presence in the array of neutral items which share visual features with the distractor, and thus dilute its effects (Tsal and Benoni, 2010; Benoni and Tsal, 2013). The paradigm described above controls for dilution as both low load and high load displays contain neutral stimuli which share a feature (shape) with the distractor triangle. Low load and high load displays are thus differentiated only by whether the target can be identified on the basis of a single feature or a conjunction of features [see Murphy and Greene (2017b) for further discussion of dilution as it pertains to this paradigm].

### Ecological Validity in a 3D World

The lateral separation of human eyes means that each has a slightly different view of the world and the disparity between these views occurs along a single horizontal dimension. This binocular disparity is greater for objects that are closer to us than those that are farther away. Using this rule, the visual system can map objects along three dimensions of the visual field; a process referred to as stereoscopic vision or stereopsis (Qian, 1997). In an exploration of the function of stereoscopic depth perception, Read (2015) points to the understanding that predatory animals acquire superior ability to detect and distinguish camouflage in visually complex environments via binocular stereopsis. The importance of this ability is underscored by the finding that human participants with normal stereoscopic vision typically out-perform counterparts with reduced or no stereoscopic vision on visuomotor skills tasks (Mazyn et al., 2004; O’Connor et al., 2010). The influential two-stream hypothesis (Goodale and Milner, 1992) provided a neurological account of vision that separated such visuospatial information processing for visually guided behaviour (dorsal stream) from slower identification or recognition processes (ventral stream). Since then, reviews and critiques have rejected the extreme claim that these visual tasks are based on functionally independent neurological processing pathways (Milner and Goodale, 2008; McIntosh and Schenk, 2009). Despite mounting evidence for considerable crosstalk between streams (Milner, 2017), there is consensus that a less extreme version of the two-stream hypothesis can be a useful model for generating hypotheses about visual performance in the real world (Schenk and McIntosh, 2010) – the world is both something to be perceived and something to be acted upon.

Stereoscopic vision (requiring two eyes) is not the only way the visual system derives spatial and depth information from a visual scene. Monocular signals (such as shade colour, occlusion, size and elevation in a plane) are common and powerful cues to depth (Howard and Rogers, 2002), allowing us to infer the spatial relations of objects in three dimensions even from two dimensional representations, such as a photograph. A limited number of previous studies within the feature integration theory paradigm have previously shown how visual search can be improved by using monocular/pictorial cues to depth (e.g., an illustration of a cube in which the faces are coloured to create the illusion of light and shade; Enns and Rensink, 1990a,b; Zhang et al., 2015). These researchers compared response times to the same set of polygons (squares and diamonds) arranged to give the impression of a 3D cube or a 2D abstract pattern, and reported that search for the visually meaningful 3D cubes was faster and less effortful than search for the 2D shapes. However, others have found that visual search was negatively affected by the inclusion of monocular (pictorial) depth cues relative to simply presenting the stimuli in 2D (Kyritsis et al., 2013). In a closely related body of work using a change blindness paradigm, inclusion of monocular depth cues of relative size, saturation and brightness to a stereoscopic display have been shown to improve visual working memory for perceptually closer items (Qian et al., 2017, 2018).

Another approach exploring depth in feature integration theory has investigated the way in which apparent distance (or visual depth plane) acts as a visual feature (Plewan and Rinkenauer, 2016, 2017). For example, a target in a search array could be identified on the basis of a single feature (its apparent location relative to the viewer) or on the basis of a conjunction of features (e.g., a red shape at a particular distance from the viewer). These studies have indicated that objects that appear to be closer to the viewer are typically responded to more quickly. Yet, in a similar paradigm, Pomplun et al. (2013) report no substantial effects of depth of plane on visual search strategy derived from eye movement data. Other work has demonstrated that the apparent proximity of stimuli affects visual tracking (Viswanathan and Mingolla, 2002) and visual working memory (He and Nakayama, 1995; Xu and Nakayama, 2007; Qian et al., 2017, 2018; Chunharas et al., 2019). Finally a third body of work has explored visual search and foraging performance in dynamic virtual reality 3D environments (Prpic et al., 2019; Kristjánsson et al., 2020), sometimes compared to traditional 2D pictorial scenes (Li et al., 2016). The value of such studies includes the interaction of visual search with memory and proprioceptive feedback from self-initiated whole body motion.

Importantly, previous research has not yet compared the effects of viewing the same stimuli in monoscopic 2D and stereoscopic 3D on visual attention, or separated those effects from the role of monocular cues to depth such as colour and shading. If stereopsis and other binocular cues to depth (e.g., binocular convergence) improve our ability to act upon the world more precisely, they may benefit performance in visually demanding experimental trials. On the other hand, if additional visual features (depth plane, shading etc.) are required to produce a 3D percept, it follows that attention must be deployed to bind these features together; this would result in slowed visual search for stimuli containing these features.

### The Present Study

In the present study we explore whether monocular and binocular cues to depth make unique demands of the visual system that limit the ecological validity of feature integration theory and perceptual load theory. We report two experiments, in which we replicated the paradigm used by Murphy and Greene (2017b) in two and three dimensions, and operationalised high and low perceptual load as conjunction and feature searches. In Experiment 1, dimensionality of the stimuli was manipulated using a combination of monocular and binocular cues to depth, while in Experiment 2 monocular and binocular cues were independently manipulated. We carried forward Murphy and Greene’s hypotheses, including that the predictions of both feature integration theory and perceptual load theory would be upheld. Specifically, feature integration theory predicts a significant interaction of search type and array size, such that reaction time would increase with set size for conjunction but not feature searches, and perceptual load theory predicts a significant interaction of perceptual load and congruency, such that distractor congruency effects would be reduced or eliminated under high perceptual load. It is unclear how the combination of monocular and binocular cues will affect search performance. While previous research suggests that depth cues offer advantages for visuospatial accuracy and visuomotor performance, results on the effects of depth on visual search identification are mixed. If the binding of features denoting depth requires attentional resources, this might constitute an additional source of perceptual load. Thus, we predict that the inclusion of depth cues will result in increased reaction times, especially in conditions where both monocular and binocular cues are provided.

## Experiment 1

### Method

#### Participants

Forty-two volunteers (17 male, 25 female) were recruited from the University College Dublin campus to participate in the experiment. Participants were aged between 18 and 52 years (*M* = 24.41 years, *SD* = 6.79 years). All had normal or corrected-to-normal vision and all were screened for colour-blindness and for stereoscopic depth perception.

#### Apparatus

The experiment was programmed and run using E-Prime 2.0 (Psychology Software Tools Inc.). A 47-inch LG Smart Cinema 3D television screen (resolution = 1920 × 1080) was used to present the stimuli in 2D or passive polarised stereoscopic 3D. For the stereoscopic 3D condition, the depth control was set to two points above the TV default, which creates a baseline crossed disparity of 12 pixels (∼0.62% of the screen width). All 3D stimuli appeared on a single depth plane with a sense of 3D space around them. To control for screen luminance effects, participants in both the 2D and 3D groups wore passive-polarised 3D glasses designed for use with this television.

#### Design

The experimental design followed Murphy and Greene (2017b) and comprised a four-way (2 × 2 × 5 × 2) factorial design with three within-subjects variables (search type: low load/feature search vs. high load/conjunction search), distractor congruency: congruent vs. incongruent, and array size: 2, 4, 6, 8, or 10 search items) and one between-subjects variable (2D vs. 3D). Participants were assigned to either the 2D or 3D condition through the use of a true randomisation technique, i.e., a coin flip (Vickers, 2006), with the exception of one participant who was assigned to the 2D group due to poor or no stereoacuity, and the final participant who was assigned to the 2D condition to ensure equal sample sizes across groups.

#### Task Stimuli

The experiment involved the search for a target stimulus within an array, while ignoring a congruent or incongruent distractor. In the 2D condition, participants were instructed to search for the target (red triangle) in an array of yellow and red shapes presented to the left of a central fixation point (a white cross), while ignoring a distractor to the right of the fixation point. Participants responded by indicating whether the target triangle was pointing up or down (using the arrows on a keyboard). In low load, feature search trials, the non-target stimuli in the search array were all yellow triangles, and the target was thus distinguished by a single feature (colour). In high load, conjunction search trials, the non-target stimuli were red squares, yellow squares and yellow triangles; a conjunction of colour (red) and shape (triangle) was thus required to distinguish the target. All shapes were created in Microsoft Office 365 ProPlus. The red shapes had an RGB value of (255, 0, 0) and the yellow shapes had an RGB value of (255, 255, 0).

**FIGURE 1:**
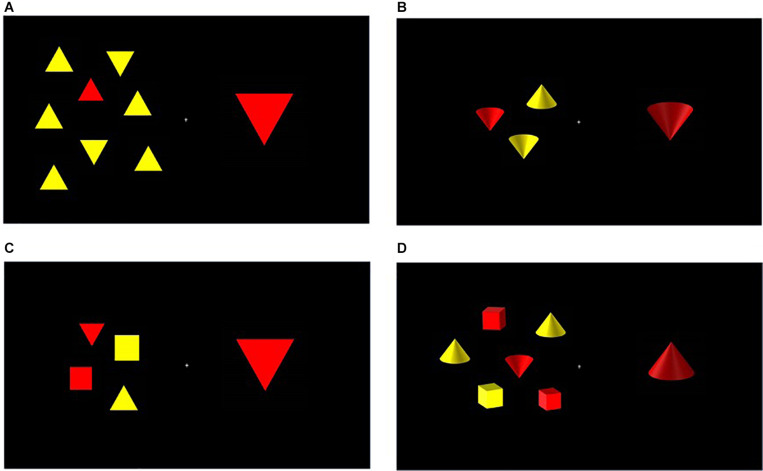
Sample trials of low load/feature (top row) and high load/conjunction (bottom row) searches in the 2D and 3D conditions in Experiment 1. **(A)** 2D Feature search. The array size is 8 and the distractor orientation is incongruent with the target. **(B)** 3D Feature search. The array size is 2 and the distractor orientation is congruent with the target. **(C)** 2D Conjunction search. The array size is 4 and the distractor orientation is congruent with the target. **(D)** 3D Conjunction search. The array size is 6 and the distractor orientation is incongruent with the target.

To supplement the binocular stereoscopic effect in the 3D condition, the above described stimuli were adapted for the 3D condition to include apparent (monocular) depth cues; this was achieved by replacing the standard two-dimensional stimuli (squares and triangles) with two-dimensional representations of three dimensional objects (cubes and cones). Shading effects were used to create the illusion of depth, as shown in Figure 1. RGB values for the front surface of the images, on which light appeared to be shining, matched those of the 2D shapes (red: 255, 0, 0; yellow: 255, 255, 0). The ‘shadowed’ surfaces of the images had RGB values ranging from (255, 5, 5) to (184, 23, 23) for red shapes and from (255, 255, 5) to (124, 124, 0) for yellow shapes. At a viewing distance of 140 cm, each shape subtended 1.44 by 1.70 degrees of visual angle. The entire search array, presented to the left of fixation, subtended between 2.1 × 3.4 (array size 2) and 6.5 × 7 degrees (array size 10). The distractor triangle, presented to the right of fixation and subtending 1.08 by 1.34 degrees, was slightly larger than the other shapes to guarantee distinctiveness and reduce eccentricity effects.

The target red triangle/cone could appear in an array of 2, 4, 6, 8, or 10 shapes and the orientation of the distractor stimulus could be congruent (i.e., both pointing up or down) or incongruent with the target (i.e., one pointing up and other pointing down). See Figure 1 for sample trials.

#### Procedure

Participants were seated 140 cm from the television screen at a desk equipped with a keyboard. After providing written consent, participants were instructed to don the 3D glasses and the task was explained to them. All lighting in the laboratory was switched off during testing. Participants first completed 30 practice trials and were given the opportunity to clarify the requirements of the task. This also allowed the experimenter to confirm that the 3D effect was perceived by participants in the 3D condition. Participants then completed three blocks of 100 trials, comprising 15 repetitions of each combination of search type, congruency and array size. Trials were presented randomly within blocks, and participants were given the opportunity to take a break following completion of each block. Participants were instructed to maintain fixation on the central white cross, and to respond to the orientation of the target stimulus as quickly and accurately as possible. The stimuli in each trial remained onscreen until a response was recorded. No performance feedback was provided.

The study protocol was approved by the Human Research Ethics Committee (Humanities) of University College Dublin.

### Results

All data are available in the Open Science Framework repository, at https://osf.io/ep5gv/. Data from one individual in the 3D condition were removed from analysis as their accuracy across all conditions was below chance (mean = 32%). Performance in the search task was generally very accurate, with mean accuracy among the remaining 41 participants of 96.5% (*SD* = 3.6%).

Median reaction time (RT) for each participant was extracted for correct search trials within each experimental condition. Median reaction time data are often preferred for such analyses as they are less susceptible than mean data to the effects of outliers (Ratcliff, 1993). A 2 (search type) × 2 (distractor congruency) × 5 (array size) × 2 (2D/3D condition) mixed within/between-subjects ANOVA was conducted. The full ANOVA results are shown in Table 1. The interested reader can find a similar analysis of accuracy data in Supplementary Tables 1, 2^1^.

**TABLE 1:** Results of the 4-way ANOVA examining effects of search type, distractor congruency, array size, and 2D/3D group on median search reaction time in Experiment 1.

Effect	*F*	*df*	*p*	η*_*p*_^2^*
**Search type**	**121.73**	**1, 39**	**<0.001**	**0.76**
Distractor congruency	0.14	1, 39	0.71	0.01
**Array size**	**115.93**	**4, 156**	**<0.001**	**0.75**
Group (2D/3D)	0.00	1, 39	0.99	0.00
Search type * distractor congruency	0.64	1, 39	0.43	0.02
Search type * array size	**41.02**	**4, 156**	**<0.001**	**0.51**
Search type * group	0.75	1, 39	0.39	0.02
Distractor congruency * array size *	0.22	4, 156	0.93	0.01
Distractor congruency * group	0.79	1, 39	0.38	0.02
Array size * group	0.35	4, 156	0.85	0.01
Search type * distractor congruency * array size	0.36	4, 156	0.55	0.01
Search type * distractor congruency * group	3.74	1, 39	0.06	0.09
Search type * array size * group	0.46	4, 156	0.77	0.01
Array size * distractor congruency * group	0.64	4, 156	0.59	0.02
Search type * distractor congruency* array size * group	0.36	4, 156	0.55	0.01

*Significant results (*p* < 0.05) are highlighted in bold.*

**FIGURE 2:**
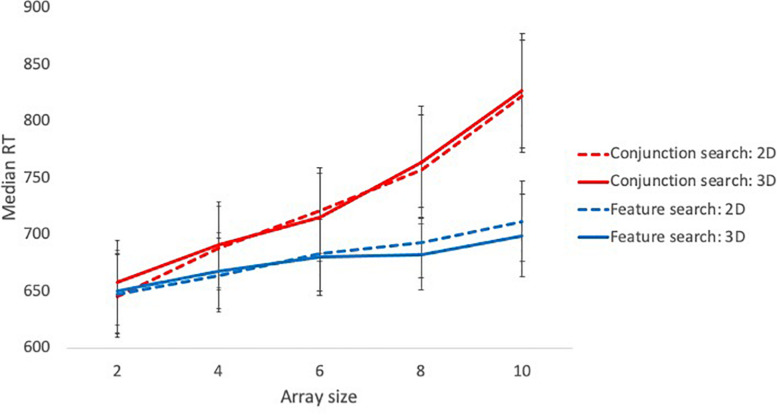
Median reaction time for feature and conjunction searches at different array sizes in Experiment 1. Error bars represent 95% confidence intervals.

A main effect of search type was observed such that responses were slower in high load/conjunction searches (*M* = 728.99 ms, *SE* = 13.66) than low load/feature searches (*M* = 677.98 ms, *SE* = 11.44). A main effect of array size was also observed; planned contrasts showed a significant linear trend, indicating that RT increased in line with the number of distractors in the array [*F*(1, 39) = 212.59, *p* < 0.001, η_p_^2^ = 0.85]. These main effects were qualified by a significant interaction, depicted in Figure 2. To investigate the predictions of feature integration theory, a linear regression of response time against array size was conducted for each search condition. Array size was a significant predictor of RT in both the conjunction search task [intercept = 603.49ms; *R*^2^ = 0.26, *F* (1, 408) = 142.5, *p* < 0.001] and the feature search task [intercept = 636.35ms; *R*^2^ = 0.06, *F* (1, 408) = 24.62, *p* < 0.001], however, the conjunction search task had a slope of 20.85 ms/item, which was significantly steeper than the feature search slope of 6.75 ms/item, *t*(816) = 6.37, *p* < 0.001. Each additional distractor in the display therefore increased reaction time by a greater magnitude in the conjunction search than the feature search.

There was no significant difference in mean reaction time between participants in the 2D (*M* = 703.4 ms, *SE* = 17.3) and 3D conditions (*M* = 703.58 ms, *SE* = 17.72). There was also no main effect of distractor congruency (congruent: *M* = 704.07 ms, *SE* = 12.68; incongruent: *M* = 702.92 ms, *SE* = 12.27) and no interaction between congruency and search type/perceptual load, as would be predicted by perceptual load theory. No other significant interaction effects were detected.

### Experiment 1 Discussion

Although the effects of array size and search type predicted by feature integration theory were observed in this experiment, no effect of distractor congruency was observed. Decades of research have reported robust congruency effects in perceptual load theory paradigms (Murphy et al., 2016), suggesting that a visible distractor cannot be easily suppressed once perceived. We therefore concluded that the distractor stimuli were not reliably perceived in this experiment. This may have been due to the peripheral placement of the distractor; Beck and Lavie (2005) have observed that distractors at fixation produce larger interference effects than distractors in the periphery. Moreover, although participants were instructed to fixate on the central fixation cross throughout, it is possible that the placement of the distractor in the periphery of a large display may have allowed participants to shift their visual field (e.g., by turning their head) and exclude the peripheral distractor from view.

In addition, no main effect of 2D/3D group, and no interaction between group and any other variable were observed in this experiment. There are two potential explanations for this finding: first, it is possible that stereoscopic depth has no bearing on reaction time during visual search, and thus has no implications for the feature integration or perceptual load models. Alternatively, the peripheral positioning of the distractor may also have undermined the stereoscopic effect produced by the LG TV used in this experiment. Although the television manufacturers claim that the 3D effect should be visible from a wide range of horizontal angles, experimenters noted that the stereoscopic effect was best viewed from directly in front of the screen and was somewhat compromised when viewing at a different angle from behind the participant. To remedy these issues, we conducted a second experiment using a centrally presented distractor, as employed in Murphy and Greene (2017b) Experiment 3.

The design of Experiment 1 did not allow the effects of monocular and binocular cues to depth to be distinguished, as flat shapes were always presented in the 2D condition and shaded shapes were always presented in the 3D condition. In order to rule out any explanation of significant results in terms of one of these factors alone, Experiment 2 therefore includes independent manipulations of stereoscopic depth (2D vs. 3D) and stimulus shading (flat vs. shaded shapes).

Finally, Experiment 1 employed a relatively small sample size and was thus somewhat underpowered. This issue is also remedied in Experiment 2.

## Experiment 2

### Method

#### Participants

One hundred and thirty participants (51 male, 79 female; age range 18–54 years; *M* = 22.85, *SD* = 6.72) were recruited from the University College Dublin campus. All participants had normal or corrected-to-normal vision, none were colour-blind, and all were screened for stereoscopic depth perception to ensure that participants allocated to the 3D condition had stereoscopic vision. Power analysis was conducted in G^∗^Power 3.1 (Faul et al., 2007). As no previous studies have compared reaction times in feature/conjunction searches between subjects in 2D and 3D conditions, no *a priori* effect size estimates were available. The sample size was therefore selected on the basis that it provided 80% power to detect medium effects (*f* = 0.25; Cohen, 1988) in the between-subjects terms. This sample also provided 80% power to detect very small effects (*f* values ranging from.03 to.06) in the within/between-subjects interaction terms; this power analysis assumed an average correlation of 0.9 among repeated measures, based on data reported in Murphy and Greene (2017b).

#### Design

This experiment employed a five-way (2 × 2 × 5 × 2 × 2) factorial design with three within-subjects variables (search type, distractor congruency, and array size, as in Experiment 1) and two between-subjects variables (stereoscopic depth: 2D/3D and stimulus shading: flat/shaded). Participants were randomly allocated to view the task in monoscopic 2D or stereoscopic 3D, and to view either flat or shaded shapes.

#### Task Stimuli

**FIGURE 3:**
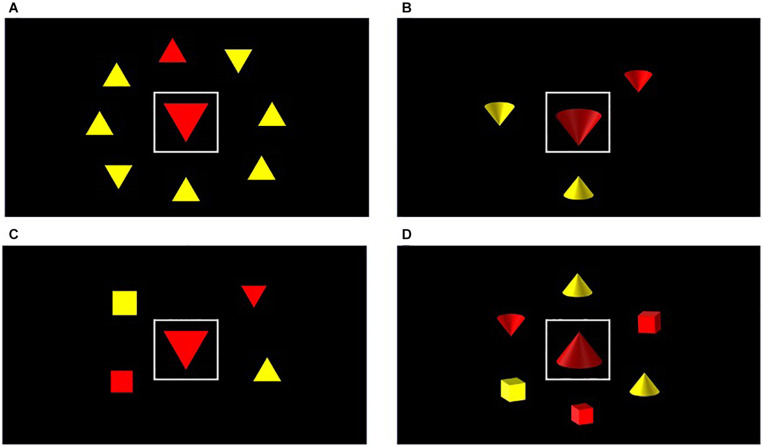
Sample trials of low load/feature [top row, panels **(A,B)**] and high load/conjunction searches [bottom row, panels **(C,D)**] in Experiment 2, using either flat [panels **(A,C)**] or shaded shapes [panels **(B,D)**]. These stimuli were displayed under either monoscopic (2D) or stereoscopic (3D) conditions.

In the task for Experiment 2, the distractor stimulus was presented in the centre of the screen surrounded by a white box, with the target appearing among an array of shapes presented in a ring around the distractor (see Figure 3). This adjusted the visual angle dimensions of the distractor which now subtended 3.83 by 3.01 degrees. The entire search array subtended 10.2 × 8.68 degrees of visual angle from a viewing distance of 140 cm.

#### Procedure

All other features of the design, task and procedure were identical to those used in Experiment 1.

### Results

All data are available in the Open Science Framework repository, at https://osf.io/ep5gv/. The data from one participant were removed from analysis due a technical error during data recording. Mean search accuracy for the remaining 129 participants was 97.3% (*SD* = 2.6%). Median RT for each participant was extracted for correct search trials within each experimental condition. Overall median RT was significantly longer in Experiment 2 (*M* = 731.94 ms, *SD* = 103.42) than Experiment 1 [*M* = 703.49, *SD* = 78.26; *t*(128) = 3.124, *p* = 0.002], suggesting that the central placement of the distractor increased the difficulty of the task.

A 2 (search type) × 2 (distractor congruency) × 5 (array size) × 2 (stereoscopic depth) × 2 (stimulus shading) ANOVA was performed. The full results of this analysis may be seen in Table 2. A similar analysis of accuracy data may be found in Supplementary Tables 3, 4^2^. A main effect of search type was observed on median reaction time, such that search was slower in high load/conjunction search trials (*M* = 766.26ms, *SE* = 10.50) than in low load/feature search trials (*M* = 697.62ms, *SE* = 7.88). A main effect of array size was also observed, such that RT increased with each additional item in the array (2 items: *M* = 681.48ms, *SE* = 7.77; 4 items: *M* = 700.08ms, *SE* = 8.14; 6 items: *M* = 725.66ms, *SE* = 8.58; 8 items: *M* = 755.64ms, *SE* = 9.75; 10 items: *M* = 794.81ms, *SE* = 10.67). Critically, there was also a significant interaction between search type and array size. Linear regression analysis showed that RT increased with array size for conjunction trials [intercept = 633.03ms; *R*^2^ = 0.44, *F*(1,648) = 154.61, *p* < 0.001, slope = 26.13 ms/item] while the slope stayed relatively flat for feature search trials [intercept = 689.36ms; *R*^2^ = 0.10, *F*(1,648) = 6.87, *p* = 0.01, slope = 3.98 ms/item]; the slope of the line was significantly steeper in the conjunction search condition, *t* (1296) = 8.54, *p* < 0.001. Thus, the predictions of feature integration theory were upheld.

**TABLE 2:** Results of the five-way ANOVA examining effects of search type, distractor congruency, array size, stereoscopic depth, and stimulus shading on median search reaction time in Experiment 2.

Effect	*F*	*df*	*p*	η*_*p*_^2^*
Main effects				
**Search type**	**394.96**	**1, 125**	**<0.001**	**0.76**
**Distractor congruency**	**115.34**	**1, 125**	**<0.001**	**0.48**
**Array size**	**333.95**	**4, 500**	**<0.001**	**0.73**
Stereoscopic depth (2D/3D)	2.43	1, 125	0.12	0.02
**Stimulus shading (flat/shaded)**	**6.34**	**1, 125**	**0.01**	**0.05**
2-way interactions				
Search type * distractor congruency	2.42	1, 125	0.12	0.02
Search type * array size	**227.78**	**4, 500**	**<0.001**	**0.65**
Search type * stereoscopic depth	3.58	1, 125	0.06	0.03
Search type * stimulus shading	**6.85**	**1, 125**	**0.01**	**0.05**
Distractor congruency * array size	1.24	4, 500	0.29	0.01
Distractor congruency * stereoscopic depth	0.06	1, 125	0.80	0.001
Distractor congruency * stimulus shading	0.002	1, 125	0.96	0.000
Array size * stereoscopic depth	**3.96**	**4, 500**	**0.004**	**0.03**
Array size * stimulus shading	1.86	4, 500	0.12	0.02
Stereoscopic depth * stimulus shading	**5.49**	**1, 125**	**0.02**	**0.04**
3-way interactions				
Search type * distractor congruency * array size	1.67	4, 500	0.15	0.01
Search type * distractor congruency * stereoscopic depth	0.005	1, 125	0.94	0.000
Search type * distractor congruency * stimulus shading	0.03	1, 125	0.86	0.000
Search type * array size * stereoscopic depth	**3.10**	**4, 500**	**0.02**	**0.02**
Search type * array size * stimulus shading	**3.03**	**4, 500**	**0.02**	**0.02**
Search type * stereoscopic depth * stimulus shading	**5.70**	**1, 125**	**0.02**	**0.04**
Array size * stereoscopic depth * stimulus shading	2.04	4, 500	0.09	0.02
Distractor congruency * stereoscopic depth * stimulus shading	1.87	1, 125	0.17	0.01
Distractor congruency * array size * stereoscopic depth	0.71	4, 500	0.69	0.01
Distractor congruency * array size * stimulus shading	1.26	4, 500	0.29	0.01
4-way interactions				
Search type * distractor congruency * array size * stereoscopic depth	1.65	4, 500	0.16	0.01
Search type * distractor congruency * array size * stimulus shading	0.74	4, 500	0.56	0.01
Search type * distractor congruency * stereoscopic depth * stimulus shading	0.88	1, 125	0.35	0.01
Search type * array size * stereoscopic depth * stimulus shading	**2.99**	**4, 500**	**0.02**	**0.02**
Distractor congruency * array size * stimulus shading * stereoscopic depth				
5-way interaction				
Search type * distractor congruency * array size * stimulus shading * stereoscopic depth	1.25	4, 500	0.29	0.01

*Significant results (*p* < 0.05) are highlighted in bold.*

**FIGURE 4:**
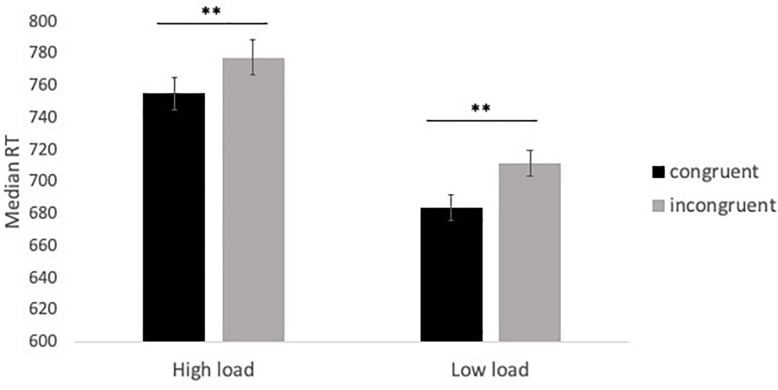
Median RT in congruent and incongruent trials under high load (conjunction search) and low load (feature search) conditions in Experiment 2. **p < 0.001.

A significant effect of distractor congruency was observed; RTs were slower in incongruent trials (*M* = 744.47 ms, *SE* = 9.33) than congruent trials (*M* = 719.41 ms, *SE* = 9.02), indicating that the distractor stimuli were perceived and interfered with search performance. However, no significant interaction between load and congruency was observed, contrary to the predictions of perceptual load theory. *Post hoc* tests indicated a significant difference in RT between congruent and incongruent trials under both low load and high load conditions, whereas perceptual load theory would predict that this effect should be eliminated or greatly reduced under high load (see Figure 4). Additional analysis indicated considerable individual differences in this effect: the predictions of load theory were upheld for approximately half the sample, with many participants demonstrating reverse congruency effects (i.e., faster RTs during congruent trials relative to incongruent trials). See Supplementary Material for further details of these analyses.

There was no main effect of stereoscopic depth (2D vs. 3D), however, a main effect of stimulus shading was observed, such that longer RTs were observed in response to shaded shapes (*M* = 753.63 ms, *SE* = 12.75) than flat shapes (*M* = 709.44 ms, *SE* = 12.06). These effects are qualified by an interaction between these two display variables. *Post hoc* Tukey tests revealed that RT in response to the 3D shaded condition (*M* = 787. 85 ms, *SE* = 17.54) was significantly higher than RT in the 3D flat (*M* = 702.56ms, *SE* = 18.75), 2D flat (*M* = 716.32, *SE* = 17.28) or 2D shaded conditions (*M* = 719.41, *SE* = 16.54, all *p*’s < 0.01); none of the other pairwise comparisons showed significant differences.

**FIGURE 5:**
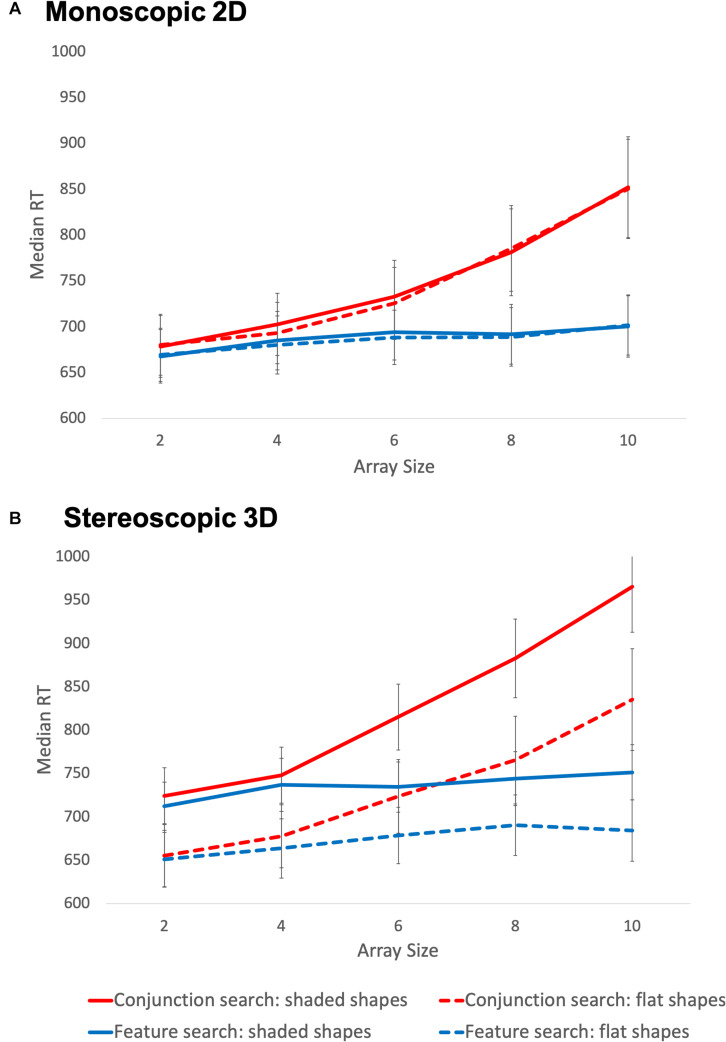
Median reaction times during Experiment 2. Panel **(A)** depicts the monoscopic 2D conditions and panel **(B)** depicts the stereoscopic 3D conditions. Error bars represent 95% confidence intervals.

A number of three-way interactions were also observed, but were qualified by a four-way interaction of search type, array size, stereoscopic depth, and stimulus shading. Figure 5 depicts the overall pattern of effects; a clear interaction between search type and array size is evident across all display conditions, such that reaction time increases steeply with array size in conjunction search conditions, but not in feature search conditions. The RTs in each cell of this analysis were remarkably similar for the 2D flat, 2D shaded and 3D flat conditions, however, in the 3D shaded condition (depicted as solid lines in Panel B), a sharp increase in overall RT was observed. Bonferroni-corrected *post hoc* tests confirmed that average RT in the 3D shaded condition was significantly higher than that in the other three conditions which did not differ significantly from one another. Thus, the same pattern of interaction between search type and array size was seen under all conditions, but this effect was moderated by the combination of monoscopic and stereoscopic cues to depth, with slower overall reaction times in the 3D shaded condition.

## Discussion

We investigated the effects of monoscopic and stereoscopic cues to depth on visual search, with the aim of validating feature integration theory and perceptual load theory in a 3D world. Across two experiments, the predictions of feature integration theory were upheld under both 2D and 3D conditions; a significant interaction of search type and array size was observed, as the slope for feature search trials remained relatively flat while reaction times in the conjunction search trials increased linearly with number of distractors. This provides support for the classic feature integration idea that the target ‘popped out’ in the low load, feature search trials, but that a serial search was required to identify the target in the high load, conjunction search trials. These data provide support for the validity of feature integration theory under more ecologically valid conditions.

In Experiment 2, this interaction of array size and search type was moderated by a combination of monoscopic and stereoscopic cues to depth. The same pattern of a steep slope in conjunction search trials and flat slope in feature search trials was observed regardless of display condition. However, overall search time was significantly increased in the condition that combined stereoscopic 3D with shaded shapes. This slowed overall response was not seen with any other combination of depth cues; stereoscopic 3D presented with flat shapes resulted in similar RTs to monoscopic 2D with either flat or shaded shapes. Thus, the addition of stereoscopic 3D and monocular cues to depth – that is, the combination of depth cues typically found in the real world – slowed overall search reaction time, but did not interfere with the typical pattern of search observed in feature integration theory studies (Quinlan, 2003; Humphreys, 2016). Feature integration theory tells us that reaction times increase in conjunction searches because attention is required to bind the features of each item together, and the items are processed serially (Treisman, 1988). The present results indicate that binding the features of stereoscopic depth and stimulus shading into a single three-dimensional percept with colour and shape required extra attention, over and above that required by the addition of either monocular or binocular depth cues alone.

The finding that there was no difference in search RT between the 2D flat and 2D shaded conditions indicates that including monocular cues to depth alone does not affect search efficiency. These results are in contrast to previous findings that visual search was negatively affected by the inclusion of monocular (pictorial) depth cues relative to simply presenting the stimuli in 2D (Kyritsis et al., 2013); they also challenge research showing that search is facilitated – i.e., faster – when the constituent polygons of a target image are presented in the form of an apparently three-dimensional object, such as a cube, rather than rearranged into an abstract shape that appears two-dimensional (Enns and Rensink, 1990a,b; Zhang et al., 2015). We suggest that these latter findings may be attributed to the semantic meaning imposed by the organisation of the polygons into a recognisable shape, rather than the representation of depth. The flat stimuli in the present experiment were composed of two features: a shape (e.g., a square) and a colour (e.g., yellow). In contrast, the shaded stimuli were comprised of multiple facets and colours – for example, the red cube had three visible faces rendered in three different red hues to denote depth via light and shade. The parallel slopes during visual search for the flat and shaded shapes suggest that, when presented in 2D, the apparently three-dimensional cube was perceived as a single shape rather than a combination of polygons.

Similarly, the absence of a difference between the 2D flat and 3D flat conditions rules out an explanation in terms of stereopsis alone, or any methodological artefact arising from the use of the 3D TV screen. Computing depth from binocular disparity and creating a coherent percept from two distinct channels of visual input is an effortful and time-consuming process (Welchman, 2016). One marked characteristic of the 3D shaded stimuli was the illusion of volume and mass. These stimuli consequently afforded the sensation of being able to “reach out and grab” the shapes, which appeared to hang in mid-air. These action affordances during observation of apparently graspable objects engage early attentional processes (Proverbio et al., 2011) and require crosstalk between ventral and dorsal streams, which is increased when stereoscopic and pictorial depth cues are combined (Verhagen et al., 2008; Milner, 2017). Thus, we suggest that the provision of both monocular and binocular cues to depth in the current study may have required additional processing time during visual search, as participants computed motor-relevant information.

The predictions of perceptual load theory were not upheld in this study: in contrast with the experiments reported by Murphy and Greene (2017b) using the same paradigm, we did not observe a significant interaction of perceptual load and distractor congruency in either experiment. No main effect of distractor congruency was observed in Experiment 1, most likely due to the peripheral placement of the distractor stimulus, however, this was addressed in Experiment 2, in which the distractor stimulus was presented centrally and the search array items were presented in a ring around the distractor. A clear distractor congruency effect was observed here, suggesting that participants did process the centrally presented distractor. However, counter to the predictions of perceptual load theory (Lavie, 2005), distractor interference was not found to be reduced under high perceptual load. As the basic predictions of perceptual load theory were not upheld in this study, we were not able to validate the model under 3D conditions. No significant interactions were observed between the critical perceptual load theory variables (perceptual load and distractor congruency) and the display variables of stereoscopic depth and stimulus shading, however, in the absence of a significant interaction of load and congruency, these results cannot be reliably interpreted. The principles of perceptual load theory have previously been extended into complex multisensory tasks, including driving (Marciano and Yeshurun, 2012, 2015; Murphy and Greene, 2015, 2016b, 2017a), playing football (Furley et al., 2013) and eyewitness memory (Murphy and Greene, 2016a; Greene et al., 2017); nevertheless, the failure to replicate the basic distractor congruency effect presents a challenge to the model and precludes confirmation of its validity in more complex environments.

Marciano and Yeshurun (2017) recently reported large interindividual and intraindividual differences in distractor perception under high and low perceptual load, suggesting that the magnitude of the perceptual load effect may be dependent on specific individual characteristics that vary at both state and trait level. Here we report similar findings; just over half of the sample in Experiment 2 showed the expected reduction in distractor interference under high load, while the remainder experienced the opposite pattern, with larger distractor congruency effects under high load than low load. Combined with Marciano and Yeshurun’s results, the findings of the current study suggest that findings from the classic perceptual load theory experiments (e.g., Lavie, 1995; Lavie et al., 2004) may not be as robust as anticipated, or at least that they are dependent on some boundary conditions such as, for example, the display or type of stimuli. One difference between the present paradigm and much of the previous perceptual load theory literature is that the distractor stimulus was presented centrally rather than in the periphery. Beck and Lavie (2005) have noted that, while perceptual load effects are observed with both centrally and peripherally placed distractors, larger distractor effects are typically observed with foveal distractors. It is possible that the distractor placement may have affected the distribution of attention to target and distractor stimuli (though note that Murphy and Greene (2017b) observed perceptual load effects in a similar paradigm with centrally resented distractors). Future research may wish to investigate these boundary conditions and identify the circumstances under which perceptual load effects can and cannot be detected.

The data presented in this paper do, however, contribute to the validity of feature integration theory as a model for evaluating visual search in a three-dimensional world. The predictions of feature integration theory were upheld by findings from both experiments in the current study. Although this was the case regardless of 2D/3D condition, longer search times were observed in conditions with a combination of monoscopic and stereoscopic depth cues. This suggests that binding features into three-dimensional objects requires greater attentional effort. In the interests of ecological validity, we call for this line of work to be extended beyond the visual domain and to consider the integration of other sensory information as a part of a multimodal process.

## Data Availability Statement

The datasets presented in this study can be found in online repositories. The names of the repository/repositories and accession number(s) can be found below: Open Science Framework, https://osf.io/ep5gv/.

## Ethics Statement

The studies involving human participants were reviewed and approved by the University College Dublin Human Research Ethics Committee (Humanities). The patients/participants provided their written informed consent to participate in this study.

## Author Contributions

CG and BR designed the study, with the assistance of JB and AH. CG programmed the experimental paradigms and conducted the statistical analysis. JB, AH, SeK, SH, and StK collected the data and assisted with analysis. CG wrote the manuscript with BR. All authors approved the final manuscript.

## Conflict of Interest

The authors declare that the research was conducted in the absence of any commercial or financial relationships that could be construed as a potential conflict of interest.

## Footnotes

1 Some significant results were observed in this analysis, including a main effect of search type, main effect of array size and an interaction between search type and distractor congruency. As these results were based on an analysis of fewer than 4% of trials, they are not commented on further here.

2 Some significant results were observed in this analysis, including main effects of search type and distractor congruency, and interactions between search type, distractor congruency and array size, As these results were based on an analysis of fewer than 3% of trials, they are not commented on further here.
