# Supplementary material for: Visual Search in 3D: Effects of Monoscopic and Stereoscopic Cues to Depth on the Validity of Feature Integration Theory and Perceptual Load Theory
Source: Front Psychol. 2021 Mar 17;12:596511. doi: 10.3389/fpsyg.2021.596511 (PMC8009999; doi:10.3389/fpsyg.2021.596511)
Supplement: Supplementary file 1 [file Table_1.DOCX]

**Visual search in 3D: Effects of monoscopic and stereoscopic cues to depth on the validity of Feature Integration Theory and Perceptual Load Theory**

**Supplemental materials**

*Effects of experimental variables on mean accuracy: Experiment 1*

**Table S-1.** Results of a 4-way ANOVA examining effects of search type, distractor congruency, array size and 2D/3D group on mean accuracy in Experiment 1.

| ***Effect*** | ***F*** | ***df*** | ***p*** | ***η_p_^2^*** |
| --- | --- | --- | --- | --- |
| **Search type** | **18.18** | **1, 39** | **< .001** | **.32** |
| Distractor congruency | 2.89 | 1, 39 | .10 | .07 |
| **Array size** | **2.90** | **4, 156** | **.02** | **.07** |
| Group (2D/3D) | 0.31 | 1, 39 | .58 | .01 |
| Search type * distractor congruency | 0.00 | 1, 39 | .99 | .000 |
| Search type * array size | 0.28 | 4, 156 | .89 | .01 |
| Search type * group | 0.02 | 1, 39 | .88 | .001 |
| Distractor congruency * array size | 1.39 | 4, 156 | .24 | .03 |
| **Distractor congruency * group** | **6.57** | **1, 39** | **.01** | **.14** |
| Array size * group | 0.38 | 4, 156 | .82 | .01 |
| Search type * distractor congruency * array size | 1.72 | 4, 156 | .15 | .04 |
| Search type * distractor congruency * group | 0.01 | 1, 39 | .94 | .000 |
| Search type * array size * group | 1.95 | 4, 156 | .11 | .05 |
| Array size * distractor congruency * group | 1.12 | 4, 156 | .35 | .03 |
| Search type * array size * distractor congruency * group | 0.22 | 4, 156 | .93 | .01 |

*Note: Significant results (p < .05) are highlighted in bold*

**Table S-2.** Mean accuracy in Experiment 1 as a function of 2D/3D group, search type, distractor congruency and array size

| ***Group*** | ***Search Type*** | ***Congruency*** | ***Array Size*** | ***Mean*** | ***S.E.*** | ***95% CI*** | |
| --- | --- | --- | --- | --- | --- | --- | --- |
|  |  |  |  |  |  | ***Lower*** | ***Upper*** |
| 2D | High load/conjunction | Congruent | 2 | 0.96 | 0.02 | 0.93 | 0.99 |
|  |  |  | 4 | 0.96 | 0.01 | 0.93 | 0.98 |
|  |  |  | 6 | 0.95 | 0.01 | 0.93 | 0.98 |
|  |  |  | 8 | 0.97 | 0.01 | 0.95 | 0.99 |
|  |  |  | 10 | 0.97 | 0.01 | 0.95 | 0.99 |
|  |  | Incongruent | 2 | 0.97 | 0.02 | 0.93 | 1.00 |
|  |  |  | 4 | 0.96 | 0.01 | 0.94 | 0.99 |
|  |  |  | 6 | 0.97 | 0.01 | 0.94 | 0.99 |
|  |  |  | 8 | 0.99 | 0.01 | 0.97 | 1.01 |
|  |  |  | 10 | 0.99 | 0.01 | 0.97 | 1.00 |
|  | Low load/feature | Congruent | 2 | 0.94 | 0.02 | 0.90 | 0.97 |
|  |  |  | 4 | 0.94 | 0.02 | 0.91 | 0.98 |
|  |  |  | 6 | 0.95 | 0.01 | 0.92 | 0.98 |
|  |  |  | 8 | 0.95 | 0.02 | 0.91 | 0.98 |
|  |  |  | 10 | 0.97 | 0.01 | 0.95 | 0.99 |
|  |  | Incongruent | 2 | 0.96 | 0.02 | 0.92 | 0.99 |
|  |  |  | 4 | 0.97 | 0.01 | 0.95 | 0.99 |
|  |  |  | 6 | 0.96 | 0.02 | 0.93 | 0.99 |
|  |  |  | 8 | 0.96 | 0.01 | 0.94 | 0.98 |
|  |  |  | 10 | 0.97 | 0.01 | 0.96 | 0.99 |
| 3D | High load/conjunction | Congruent | 2 | 0.98 | 0.02 | 0.95 | 1.01 |
|  |  |  | 4 | 0.98 | 0.01 | 0.95 | 1.00 |
|  |  |  | 6 | 0.97 | 0.01 | 0.95 | 1.00 |
|  |  |  | 8 | 0.98 | 0.01 | 0.96 | 1.00 |
|  |  |  | 10 | 0.98 | 0.01 | 0.95 | 1.00 |
|  |  | Incongruent | 2 | 0.95 | 0.02 | 0.92 | 0.99 |
|  |  |  | 4 | 0.98 | 0.01 | 0.95 | 1.01 |
|  |  |  | 6 | 0.97 | 0.01 | 0.95 | 0.99 |
|  |  |  | 8 | 0.98 | 0.01 | 0.96 | 1.00 |
|  |  |  | 10 | 0.98 | 0.01 | 0.97 | 1.00 |
|  | Low load/feature | Congruent | 2 | 0.97 | 0.02 | 0.94 | 1.01 |
|  |  |  | 4 | 0.93 | 0.02 | 0.90 | 0.97 |
|  |  |  | 6 | 0.97 | 0.01 | 0.94 | 0.99 |
|  |  |  | 8 | 0.98 | 0.02 | 0.95 | 1.01 |
|  |  |  | 10 | 0.97 | 0.01 | 0.95 | 0.99 |
|  |  | Incongruent | 2 | 0.96 | 0.02 | 0.93 | 0.99 |
|  |  |  | 4 | 0.97 | 0.01 | 0.95 | 0.99 |
|  |  |  | 6 | 0.94 | 0.02 | 0.91 | 0.97 |
|  |  |  | 8 | 0.97 | 0.01 | 0.95 | 0.99 |
|  |  |  | 10 | 0.97 | 0.01 | 0.95 | 0.98 |

*Effects of experimental variables on mean accuracy: Experiment 2*

**Table S-3.** Results of a five-way ANOVA examining effects of search type, distractor congruency, array size, stereoscopic depth and stimulus shading on mean accuracy in Experiment 2.

|  | ***Effect*** | ***F*** | ***df*** | ***p*** | ***η_p_^2^*** |
| --- | --- | --- | --- | --- | --- |
| *Main effects* | |  |  |  |  |
|  | **Search type** | **15.71** | **1, 125** | **< .001** | **.11** |
|  | **Distractor congruency** | **57.17** | **1, 125** | **< .001** | **.31** |
|  | Array size | 1.42 | 4, 500 | .23 | .01 |
|  | Stereoscopic depth (2D/3D) | 0.17 | 1, 125 | .68 | .001 |
|  | Stimulus shading (flat/shaded) | .001 | 1, 125 | .97 | .000 |
| *2-way interactions* | |  |  |  |  |
|  | **Search type * distractor congruency** | **4.21** | **1, 125** | **.04** | **.03** |
|  | **Search type * array size** | **3.73** | **4, 500** | **.005** | **.03** |
|  | Search type * stereoscopic depth | 0.12 | 1, 125 | .72 | .001 |
|  | Search type * stimulus shading | .08 | 1, 125 | .77 | .001 |
|  | Distractor congruency * array size | 1.23 | 4, 500 | .30 | .01 |
|  | Distractor congruency * stereoscopic depth | 1.17 | 1, 125 | .28 | .01 |
|  | Distractor congruency * stimulus shading | 0.18 | 1, 125 | .67 | .001 |
|  | Array size * stereoscopic depth | 0.23 | 4, 500 | .92 | .002 |
|  | Array size * stimulus shading | 0.55 | 4, 500 | .70 | .004 |
|  | Stereoscopic depth * stimulus shading | 1.71 | 1, 125 | .19 | .01 |
| *3-way interactions* | |  |  |  |  |
|  | **Search type * distractor congruency * array size** | **2.40** | **4, 500** | **.05** | **.02** |
|  | Search type * distractor congruency * stereoscopic depth | 1.80 | 1, 125 | .18 | .01 |
|  | Search type * distractor congruency * stimulus shading | 0.02 | 1, 125 | .89 | .000 |
|  | Search type * array size * stereoscopic depth | 0.53 | 4, 500 | .71 | .004 |
|  | **Search type * array size * stimulus shading** | **2.77** | **4, 500** | **.03** | **.02** |
|  | Search type * stereoscopic depth * stimulus shading | 0.002 | 1, 125 | .96 | .000 |
|  | Array size * stereoscopic depth * stimulus shading | 0.76 | 4, 500 | .55 | .01 |
|  | Distractor congruency * stereoscopic depth * stimulus shading | 1.87 | 1, 125 | .17 | .01 |
|  | Distractor congruency * array size * stereoscopic depth | 0.83 | 4, 500 | .51 | .01 |
|  | Distractor congruency * array size * stimulus shading | 1.10 | 4, 500 | .36 | .01 |
| *4-way interactions* | |  |  |  |  |
|  | Search type * distractor congruency * array size * stereoscopic depth | 0.34 | 4, 500 | .84 | .003 |
|  | Search type * distractor congruency * array size * stimulus shading | 0.71 | 4, 500 | .58 | .01 |
|  | Search type * distractor congruency * stereoscopic depth * stimulus shading | 0.48 | 1, 125 | .49 | .004 |
|  | Search type * array size * stereoscopic depth * stimulus shading | 0.89 | 4, 500 | .47 | .01 |
|  | Distractor congruency * array size * stimulus shading * stereoscopic depth | 0.26 | 4, 500 | .90 | .002 |
| *5-way interaction* | |  |  |  |  |
|  | Search type * distractor congruency * array size * stimulus shading * stereoscopic depth | 0.69 | 4, 500 | .59 | .005 |

*Note: Significant results (p < .05) are highlighted in bold*

**Table S-4.** Mean accuracy in Experiment 2 as a function of stimulus shading, stereoscopic depth, search type, distractor congruency and array size

| ***Stimulus shading*** | ***Stereoscopic depth*** | ***Search type*** | ***Congruency*** | ***Array size*** | ***Mean*** | ***SE*** | ***95% CI*** | |
| --- | --- | --- | --- | --- | --- | --- | --- | --- |
|  |  |  |  |  |  |  | ***Lower*** | ***Upper*** |
| flat | 2D | High load/conjunction | Congruent | 2 | 0.97 | 0.01 | 0.96 | 0.99 |
|  |  |  |  | 4 | 0.97 | 0.01 | 0.96 | 0.98 |
|  |  |  |  | 6 | 0.98 | 0.01 | 0.97 | 0.99 |
|  |  |  |  | 8 | 0.98 | 0.01 | 0.97 | 0.99 |
|  |  |  |  | 10 | 0.99 | 0.01 | 0.98 | 1.01 |
|  |  |  | Incongruent | 2 | 0.96 | 0.01 | 0.93 | 0.98 |
|  |  |  |  | 4 | 0.95 | 0.01 | 0.93 | 0.97 |
|  |  |  |  | 6 | 0.97 | 0.01 | 0.95 | 0.99 |
|  |  |  |  | 8 | 0.98 | 0.01 | 0.96 | 0.99 |
|  |  |  |  | 10 | 0.98 | 0.01 | 0.97 | 0.99 |
|  |  | Low load/feature | Congruent | 2 | 0.98 | 0.01 | 0.97 | 0.99 |
|  |  |  |  | 4 | 0.98 | 0.01 | 0.96 | 0.99 |
|  |  |  |  | 6 | 0.98 | 0.01 | 0.96 | 0.99 |
|  |  |  |  | 8 | 0.98 | 0.01 | 0.97 | 1.00 |
|  |  |  |  | 10 | 0.98 | 0.01 | 0.96 | 0.99 |
|  |  |  | Incongruent | 2 | 0.96 | 0.01 | 0.94 | 0.98 |
|  |  |  |  | 4 | 0.95 | 0.01 | 0.94 | 0.97 |
|  |  |  |  | 6 | 0.96 | 0.01 | 0.94 | 0.98 |
|  |  |  |  | 8 | 0.95 | 0.01 | 0.92 | 0.97 |
|  |  |  |  | 10 | 0.93 | 0.01 | 0.91 | 0.96 |
|  | 3D | High load/conjunction | Congruent | 2 | 0.98 | 0.01 | 0.97 | 1.00 |
|  |  |  |  | 4 | 0.98 | 0.01 | 0.97 | 1.00 |
|  |  |  |  | 6 | 0.98 | 0.01 | 0.97 | 1.00 |
|  |  |  |  | 8 | 0.99 | 0.01 | 0.97 | 1.00 |
|  |  |  |  | 10 | 0.99 | 0.01 | 0.98 | 1.01 |
|  |  |  | Incongruent | 2 | 0.97 | 0.01 | 0.95 | 1.00 |
|  |  |  |  | 4 | 0.97 | 0.01 | 0.95 | 0.99 |
|  |  |  |  | 6 | 0.97 | 0.01 | 0.96 | 0.99 |
|  |  |  |  | 8 | 0.98 | 0.01 | 0.96 | 0.99 |
|  |  |  |  | 10 | 0.99 | 0.01 | 0.98 | 1.00 |
|  |  | Low load/feature | Congruent | 2 | 0.98 | 0.01 | 0.97 | 1.00 |
|  |  |  |  | 4 | 0.98 | 0.01 | 0.96 | 0.99 |
|  |  |  |  | 6 | 0.98 | 0.01 | 0.97 | 1.00 |
|  |  |  |  | 8 | 0.98 | 0.01 | 0.96 | 0.99 |
|  |  |  |  | 10 | 0.98 | 0.01 | 0.96 | 1.00 |
|  |  |  | Incongruent | 2 | 0.96 | 0.01 | 0.94 | 0.98 |
|  |  |  |  | 4 | 0.98 | 0.01 | 0.96 | 1.00 |
|  |  |  |  | 6 | 0.98 | 0.01 | 0.96 | 1.00 |
|  |  |  |  | 8 | 0.96 | 0.01 | 0.94 | 0.99 |
|  |  |  |  | 10 | 0.96 | 0.01 | 0.93 | 0.99 |
| shaded | 2D | High load/conjunction | Congruent | 2 | 0.99 | 0.01 | 0.97 | 1.00 |
|  |  |  |  | 4 | 0.98 | 0.01 | 0.97 | 1.00 |
|  |  |  |  | 6 | 0.99 | 0.01 | 0.98 | 1.00 |
|  |  |  |  | 8 | 0.99 | 0.01 | 0.98 | 1.00 |
|  |  |  |  | 10 | 0.98 | 0.01 | 0.97 | 1.00 |
|  |  |  | Incongruent | 2 | 0.96 | 0.01 | 0.94 | 0.98 |
|  |  |  |  | 4 | 0.98 | 0.01 | 0.96 | 1.00 |
|  |  |  |  | 6 | 0.97 | 0.01 | 0.95 | 0.98 |
|  |  |  |  | 8 | 0.98 | 0.01 | 0.97 | 1.00 |
|  |  |  |  | 10 | 0.98 | 0.01 | 0.97 | 0.99 |
|  |  | Low load/feature | Congruent | 2 | 0.98 | 0.01 | 0.97 | 1.00 |
|  |  |  |  | 4 | 0.98 | 0.01 | 0.96 | 0.99 |
|  |  |  |  | 6 | 0.98 | 0.01 | 0.96 | 0.99 |
|  |  |  |  | 8 | 0.98 | 0.01 | 0.96 | 0.99 |
|  |  |  |  | 10 | 0.99 | 0.01 | 0.97 | 1.00 |
|  |  |  | Incongruent | 2 | 0.96 | 0.01 | 0.95 | 0.98 |
|  |  |  |  | 4 | 0.97 | 0.01 | 0.95 | 0.98 |
|  |  |  |  | 6 | 0.96 | 0.01 | 0.94 | 0.98 |
|  |  |  |  | 8 | 0.96 | 0.01 | 0.94 | 0.98 |
|  |  |  |  | 10 | 0.95 | 0.01 | 0.93 | 0.98 |
|  | 3D | High load/conjunction | Congruent | 2 | 0.98 | 0.01 | 0.97 | 1.00 |
|  |  |  |  | 4 | 0.98 | 0.01 | 0.96 | 0.99 |
|  |  |  |  | 6 | 0.99 | 0.01 | 0.98 | 1.00 |
|  |  |  |  | 8 | 0.99 | 0.01 | 0.98 | 1.00 |
|  |  |  |  | 10 | 0.97 | 0.01 | 0.96 | 0.99 |
|  |  |  | Incongruent | 2 | 0.95 | 0.01 | 0.93 | 0.97 |
|  |  |  |  | 4 | 0.97 | 0.01 | 0.95 | 0.99 |
|  |  |  |  | 6 | 0.97 | 0.01 | 0.96 | 0.99 |
|  |  |  |  | 8 | 0.97 | 0.01 | 0.95 | 0.98 |
|  |  |  |  | 10 | 0.98 | 0.01 | 0.96 | 0.99 |
|  |  | Low load/feature | Congruent | 2 | 0.98 | 0.01 | 0.96 | 0.99 |
|  |  |  |  | 4 | 0.97 | 0.01 | 0.95 | 0.98 |
|  |  |  |  | 6 | 0.98 | 0.01 | 0.96 | 0.99 |
|  |  |  |  | 8 | 0.99 | 0.01 | 0.97 | 1.00 |
|  |  |  |  | 10 | 0.98 | 0.01 | 0.96 | 0.99 |
|  |  |  | Incongruent | 2 | 0.96 | 0.01 | 0.95 | 0.98 |
|  |  |  |  | 4 | 0.96 | 0.01 | 0.94 | 0.97 |
|  |  |  |  | 6 | 0.95 | 0.01 | 0.93 | 0.97 |
|  |  |  |  | 8 | 0.96 | 0.01 | 0.94 | 0.98 |
|  |  |  |  | 10 | 0.96 | 0.01 | 0.93 | 0.98 |

*Predictions of perceptual load theory and individual differences*

Perceptual load theory states that high perceptual load reduces distractor perception, and therefore predicts that distractor congruency effects (RT for incongruent trials – RT for congruent trials) should be significantly smaller under high perceptual load compared with low perceptual load. Recent research has however reported large individual differences in the effect of perceptual load on distractor perception, with many participants displaying effects opposite to those predicted by the theory (Marciano & Yeshurun, 2017). The mean difference in congruency effect between the low load (feature search) and high load (conjunction search) conditions in the current experiment was 5.59 ms, with a standard deviation of 40.66 ms, indicating substantial variation in the effect. Figure S-1 shows the difference in distractor congruency effects between feature search and conjunction search trials for every participant in Experiment 2. Load theory predicts that the magnitude of the congruency effect should be larger for low load, feature search trials, and so the mean difference for each participant should be positive. The predictions of load theory were upheld for 75 participants (58.1%) who showed a difference in congruency effect greater than zero. For the remaining 41.9% of participants, congruency effects were found to be larger in the low load than the high load condition. This variation in the effects of perceptual load was observed regardless of display condition (monoscopic 2D or stereoscopic 3D, flat or shaded shapes). Moreover, for approximately 13% of the sample (17 out of 129 participants), the absolute magnitude of the difference in congruency effect between conjunction and feature search trials was less than 5ms, indicating no substantial difference in distractor perception between low load and high load conditions.


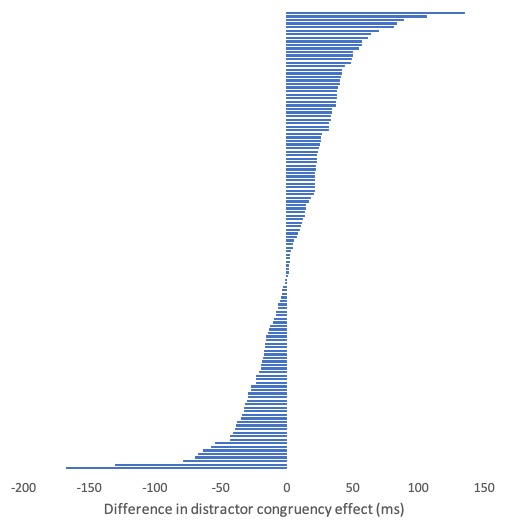


**Figure S-1.** Difference in distractor congruency effect (ms) between low load (feature search) and high load (conjunction search) trials for each participant in Experiment 2. Positive values indicate a stronger effect under low load than high load, as predicted by perceptual load theory.

**References**

Marciano, H., & Yeshurun, Y. (2017). Large inter-individual and intra-individual variability in the effect of perceptual load. PLoS One, 12(4), e0175060.
